# Economic evaluations of mammography to screen for breast cancer in low- and middle-income countries: A systematic review

**DOI:** 10.7189/jogh.12.04048

**Published:** 2022-07-16

**Authors:** Ajeng V Icanervilia, Jurjen van der Schans, Qi Cao, Adriana C de Carvalho, Kathya Cordova-Pozo, Jarir At Thobari, Maarten J Postma, Antoinette DI van Asselt

**Affiliations:** 1 Department of Health Sciences, University of Groningen, University Medical Center Groningen, Groningen, the Netherlands; 2 Department of Radiology, Faculty of Medicine, Public Health and Nursing, Universitas Gadjah Mada, Yogyakarta, Indonesia; 3 Clinical Epidemiology and Biostatistics Unit (CEBU), Faculty of Medicine, Public Health and Nursing, Universitas Gadjah Mada, Yogyakarta, Indonesia; 4 Department of Economics, Econometrics & Finance, University of Groningen, Faculty of Economics & Business, Groningen, the Netherlands; 5 Regenerative Medicine Center Utrecht, University Medical Centre Utrecht, Utrecht, the Netherlands; 6 Institute of Management Research, Radboud University, the Netherlands; 7 Department of Pharmacology and Therapy, Faculty of Medicine, Public Health and Nursing, Universitas Gadjah Mada, Yogyakarta, Indonesia; 8 Department of Pharmacology & Therapy, Universitas Airlangga, Surabaya, Indonesia; 9 Center of Excellence in Higher Education for Pharmaceutical Care Innovation, Universitas Padjadjaran, Bandung, Indonesia; 10 Department of Epidemiology, University of Groningen, University Medical Center Groningen, Groningen, the Netherlands

## Abstract

**Background:**

Low- and middle-income countries (LMICs) have limited resources compared to high-income countries (HICs). Therefore, it is critical that LMICs implement cost-effective strategies to reduce the burden of breast cancer. This study aimed to answer the question of whether mammography is a cost-effective breast cancer screening method in LMICs.

**Methods:**

A systematic article search was conducted through Medline, Embase, Web of Science, and Econlit. Studies were included only if they conducted a full economic evaluation and focused on mammography screening in LMICs. Two reviewers screened through the title and abstract of each article and continued with full-text selection. Data were extracted and synthesized narratively. Quality assessment for each included study was conducted using the Consensus Health Economic Criteria (CHEC) extended checklist.

**Results:**

This review identified 21 studies economically evaluating mammography as a breast cancer screening method in LMICs. Eighteen of these studies concluded that mammography screening was a cost-effective strategy. Most studies (71%) were conducted in upper-middle-income countries (Upper MICs). The quality of the studies varied from low to good. Important factors determining cost-effectiveness are the target age group (eg, 50-59 years), the screening interval (eg, biennial or triennial), as well as any combination with other breast cancer control strategies (eg, combination with treatment strategy for breast cancer patients).

**Conclusions:**

Mammography screening appeared to be a cost-effective strategy in LMICs, particularly in Upper MICs. More studies conducted in lower-middle-income and low-income countries are needed to better understand the cost-effectiveness of mammography screening in these regions.

Breast cancer is a significant public health problem affecting millions of women worldwide [1]. The health and economic burdens of breast cancer remain high, despite multiple attempts to resolve them (ie, lifestyle modification, chemoprevention) [2-4]. Initially considered an issue only in high-income countries (HICs), most new cancer cases occurred in low- and middle-income countries (LMICs) over the last decades. In LMICs, mortality-to-incidence ratios for breast cancer are worse than in HICs. The mortality-to-incidence ratios in Middle, Eastern, and West Africa are as high as 0.55, compared with 0.16 in North America. It means breast cancer patients are almost 3.5 times more likely to die from this disease in LMICs than in HICs [5].

An important reason behind unfavorable clinical outcomes for breast cancer patients in LMICs is that it tends to be diagnosed at a later stage [6], which may be caused by a lack of possibilities for early detection [7,8]. Screening as an early detection method is key in minimizing public health burden [9,10]. The WHO and other international cancer networks suggest that mammography screening is successful to reduce mortality and it remains the gold standard for breast cancer screening [1,11,12]. At least 30 countries worldwide, most of them HICs, have implemented a population-based mammography screening program for women [13,14].

However, such programs should not be adopted hastily in LMICs, as most of them have a weak health infrastructure and rely on limited health care resources. Thus, it is critical that they implement the most cost-effective screening method [15-17]. A 2014 WHO position paper on mammography screening [1] stated that mammography screening was a cost effective strategy in upper-middle-income countries (Upper MICs), but it was not cost-effective in a lower-middle-income country (Lower MIC). However, this statement was based on only three studies in LMICs conducted in 2012-2013. A review of systematic reviews published in 2019 explored the cost-effectiveness of breast cancer screening worldwide [18] but included mostly systematic reviews from high-income settings and could not make a clear conclusion about the cost-effectiveness of breast cancer screening in LMICs.

Therefore, it is essential to demonstrate which breast cancer screening strategies are cost-effective in LMICs. Our study reviewed all published evidence up-to-date to answer whether mammography, with or without other breast control strategies, is a cost-effective breast cancer screening tool in LMICs.

## METHODS

The review protocol for his study was published in the International Prospective Register of Systematic Reviews (PROSPERO), registration number CRD42019133781 [19]. The reporting was developed following PRISMA guidelines [20].

### Search strategy

In this study, we systematically searched MEDLINE, EMBASE, EconLit, and Web of Science on January 30, 2020. We updated the search on June 16, 2021, and October 20, 2021. These are the four main databases recommended by van Mastrigt et al. for searching economic evaluation studies [21]. The search strategies were adapted for each database, which included the terms relating to or describing the population, intervention, and study design of our review, as follow: “breast cancer” AND “mammography” AND “screening” AND “economic evaluation” AND “LMIC”. The full search strategy is available upon request. Advanced search and “exploding” a search term for retrieving all records were done with not only the exact term, but also those in the hierarchy of the controlled vocabulary using medical subject headings (MESH) in Pubmed and EMTREE in EMBASE. We did not limit the search by language or year. The search was also expanded by identifying studies from the reference lists of identified relevant studies.

### Studies selection and eligibility criteria

Articles found from our search were merged in a reference manager (Mendeley) to check and remove duplicates. After that, two reviewers (AVI, JVS) screened the title and abstract of each article. We used Abstrackr online tool to upload and organize the title and abstracts of search results for a systematic review [22]. Undecided results were still included for the next step. Then, the full texts of remaining articles were assessed for eligibility by two reviewers (AVI, JVS). If disputes about exclusion/inclusion occurred, a third reviewer was consulted (ADIA) to reach consensus.

Studies were included if they fit the following PICOS eligibility criteria: 1) Population: breast cancer and low- and middle- income countries (LMICs), as defined by the 2019 World Bank LMIC list; 2) Intervention: mammography for screening purpose; 3) Comparison: no screening or other breast cancer control programs; 4) Outcome: incremental cost-effectiveness ratio (ICER) or average cost-effectiveness ratio (ACER); 5) Study design: full economic evaluations (ie, cost-effectiveness, cost-utility, cost-benefit, cost-minimization) [23]. We excluded studies with no available full text as well as studies about mammography for diagnostic or treatment purposes (when not for population screening) and studies on interventions to improve mammography screening rates.

### Extracted information

We extracted the following characteristics from the included studies: country or region (including LMIC classification), objective, study population, intervention, and comparison. We also documented the following methodological characteristics: type of economic evaluation (eg, cost-effectiveness analysis, cost-utility analysis, other types), study design (eg, experimental, observational, model-based, etc.), perspective, time horizon, currency, currency year, and outcome measure for effectiveness, such as disability-adjusted life years (DALY), quality-adjusted life years (QALY), life-years gained (LYG) and intermediate outcome measures.

Additionally, we documented the following details: discount rates, reported incremental analysis (incremental effectiveness, incremental cost, average cost-effectiveness ratio, incremental cost-effectiveness ratio), and type of applied sensitivity analysis (one-way sensitivity analysis, probabilistic sensitivity analysis, scenario analysis). ACER is defined by the average of cost per effect, while ICER is the ratio of the change in cost to the change in effect [24]. We also displayed the main conclusion of each study.

### Study evaluation

Five reviewers (AVI, ADI, QC, KCP, ACC) assessed the risk of bias for each included study using the recommended approach, the Consensus Health Economic Criteria (CHEC) extended checklist [25-27]. This 20-item checklist can appraise model-based or trial-based studies, with positive responses scored 1 and negative responses scored 0 [21]. The total score for each item was summed and converted to a percentage with the final scores ranging from zero to 100 (final score = [total score/20] × 100%). The total CHEC score for each study was categorized into four grades based on cut-off values: low (≤50), moderate (51-75), good (76-95), and excellent (>95). Higher scores denote higher quality. We also presented the results in a graph using the Review Manager 5.4 software. The list of the 20 individual items assessed is presented in **Table 1**.

**Table 1:** List of questions assessed in CHEC-extended checklist [25]

No	Questions
1	Is the study population clearly described?
2	Are competing alternatives clearly described?
3	Is a well-defined research question posed in answerable form?
4	Is the economic study design appropriate to the stated objective?
5	Are the structural assumptions and the validation methods of the model properly reported?
6	Is the chosen time horizon appropriate in order to include relevant costs and consequences?
7	Is the actual perspective chosen appropriate?
8	Are all important and relevant costs for each alternative identified?
9	Are all costs measured appropriately in physical units?
10	Are costs valued appropriately?
11	Are all important and relevant outcomes for each alternative identified?
12	Are all outcomes measured appropriately?
13	Are outcomes valued appropriately?
14	Is an appropriate incremental analysis of costs and outcomes of alternatives performed?
15	Are all future costs and outcomes discounted appropriately?
16	Are all important variables, whose values are uncertain, appropriately subjected to sensitivity analysis?
17	Do the conclusions follow from the data reported?
18	Does the study discuss the generalizability of the results to other settings and patient/client groups?
19	Does the article/report indicate that there is no potential conflict of interest of study researcher(s) and funder(s)?
20	Are ethical and distributional issues discussed appropriately?

## RESULTS

### Search results

**Figure 1:**
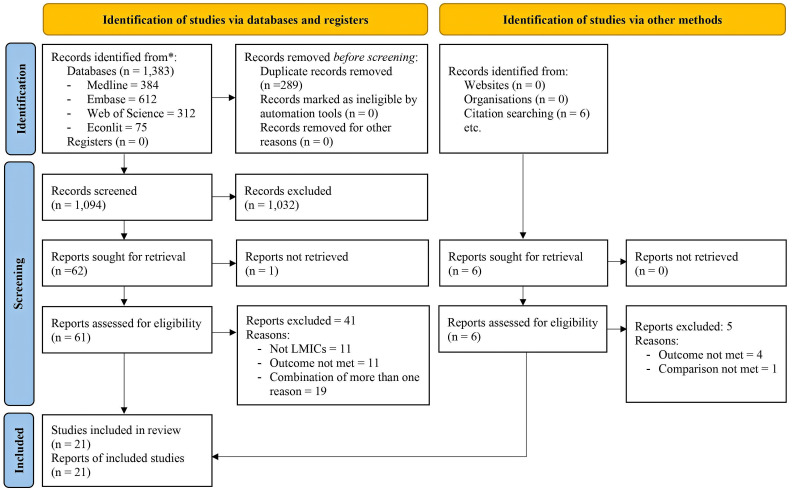
PRISMA (2020) flow diagram [20].

The stepwise selections of articles according to our selection criteria are presented in **Figure 1**. Our search process resulted in 1389 studies: 384 studies from MEDLINE, 612 studies from EMBASE, 312 studies from Web of Science, 75 studies from EconLit, and six studies identified from the reference lists of identified relevant studies. By merging the results of all individual search strategies and excluding duplicates, the total number of hits was reduced to 1100 studies. Upon screening titles and abstracts and full texts, we identified 21 articles that met our inclusion criteria.

### Study characteristics

**Table 2** describe the characteristics of 21 included studies. Among all 21 studies from LMICs, 16 studies were from Upper MICs, 3 studies were from Lower MICs, and 2 studies were from both MIC and Low-Income Countries (LICs). We found nine studies from Asia [28-32,34,35,37,47], two studies from Africa [43,48], one study from both Africa and Asia (undetermined which country) [42], five studies from South America [39-41,44,45], two studies from North America [36,46], one study from both South and North America (Mexico and Costa Rica) [38], and one study from Europe (Turkey) [33].

**Table 2:** Characteristics of reviewed studies

Authors	Country (level of income)	Study design	Economic evaluation type	Population	Interventions compared
**Wang, 2021** [28]	China (Upper MIC)	Model based	CUA	Women aged 40-70 years in China	Eight mammography screening strategies VS no screening: biennial (45-70 years; 40-70 years; 40-65 years; 50-70 years) and triennial (45-70 years; 40-70 years; 40-65 years; 50-70 years) – coverage rate 100%, 80%, or 60%
**Salikhanov, 2019** [29]	Kazakhstan (Upper MIC)	Observational	CUA	Women aged 50-60 years old in Kazakhstan	Mammography screening VS no screening
**Sun, 2018** [30]	China (Upper MIC)	Model based	CUA	Women aged 45-69 years and high risk for breast cancer in China	(1). Risk-based screening program (combination of high risk women, ultrasound and mammography) VS no screening (2). Combination of ultrasound and mammography VS mammography only
**Nguyen, 2018** [31]	Vietnam (Lower MIC)	Model based	CEA	Women aged 45-64 years old in Vietnam	Mammography screening VS no screening
**Wu, 2017** [32]	China (Upper MIC)	Model based	CUA	Women aged 45-85 years old in Shanghai, China	Twelve biennial screening strategies VS no screening Three single (MMG only; CBE only; US only); 4 in parallel (CBE + US, CBE + MMG, US + MMG, CBE + US + MMG); 4 in series (CBE-US, CBE-MMG, US-MMG, CBE-US-MMG); 1 mixed (CBE + US-MMG)
**Ozmen, 2017** [33]	Turkey (Upper MIC)	Observational	CEA	Women aged 40-69 years old in Turkey	BSMP (three biennial mammography) VS TNBCRP (no screening)
**Zehtab, 2016** [34]	Iran (Upper MIC)	Model based	CEA	Women aged 35-69 years old in rural area of Kerman, Iran	Mammography screening VS no screening
**Haghighat, 2016** [35]	Iran (Upper MIC)	Model based	CUA	Women aged 40-70 years old in Iran	Mammography screening VS no screening
**Ulloa-Pérez, 2016** [36]	Mexico (Upper MIC)	Model based	CEA	Women aged 25-75 years old in Mexico	Mammography screening VS no screening
**Barfar,2014** [37]	Iran (Upper MIC)	Observational	CEA	Women aged 35 years and higher in Iran	Mammography screening VS no screening
**Nie ¨ns, 2014** [38]	Costa Rica and Mexico (Upper MIC)	Model based	CEA	Women aged 40-70 years old in Costa Rica and Mexico	*19 breast control strategies, with four predominant strategies in Costa Rica*: (1) Current country specific situation (80%); (12) Biennial mammography screening (age 40–70) + treatment of stage I to IV (95%); (13) Biennial mammography screening (age 40–70) + treatment of stage I to IV + Trastuzumab (95%); (15) Biennial CBE screening (age 40–70) +treatment of stage I to IV (95%). *19 breast control strategies, with four predominant strategies in Mexico*: (10) Biennial mammography screening (age 50-70) + treatment of stage I to IV (95%); (11) Biennial mammography screening (age 50-70) + treatment of stage I to IV + Trastuzumab (95%); (13) Biennial mammography screening (age 40-70) + treatment of stage I to IV + Trastuzumab (95%); (14) Basic awareness outreach program + MAR + treatment of stage I to IV (95%)
**Souza, 2013** [39]	Brazil (Upper MIC)	Model based	CUA	Women aged 40-69 years old in Brazil	(A) Usual care (base); (B) SFM annual; (C) SFM every 2 years; (D) FFDM annual; (E) FFDM every 2 years; (F) FFDM age <50 and SFM age 50-69 annual; (G) FFDM annual age <50 and SFM age 50-69 every 2 years
**Zelle, 2013** [40]	Peru (Upper MIC)	Model based	CEA	Women aged 40-69 years old in Peru	*94 breast control strategies with four predominant strategies*: (59) Stage I to IV treatment with biennial mammography screening (age 40-69) fixed 60%/mobile 40%*; (65) Stage I to IV treatment with triennial mammography screening (age 40-69) fixed 60%/mobile 40%*; (67) Stage I to IV treatment with triennial mammography screening (age 45-69) fixed 60%/mobile 40%*; (94) Stage I to IV treatment with most expensive screening strategy (annual, fixed 60%/ mobile 40%, age 40-69) + EPC + trastuzumab (all stages)
**Ribeiro, 2013** [41]	Brazil (Upper MIC)	Model based	CUA	Women aged 40-69 years old in Brazil	A) Usual care vs B) NMPOA: Annual mammography screening; clinical breast examination; risk factors assessment (including genetic risk) and active search of participants by community health workers
**Ginsberg, 2012** [42]	Sub-Saharan Africa (very high adult and child mortality) and South East Asia (high adult and child mortality) – (undetermined, most likely Lower MIC and LIC)	Model based	CEA	Women aged 50-70 years old in Sub-Saharan Africa and South East Asia	*Breast cancer control strategies with three predominant strategies*: (6) 50% coverage of optimal programme (treatment of stages I-IV cancer plus biennial mammography screening); (12) 80% coverage of optimal programme; (18) 95% coverage of optimal programme
**Zelle, 2012** [43]	Ghana (Lower MIC)	Model based	CEA	Women aged 40-69 years in Ghana	*17 breast control strategies with three predominant strategies*: (11) Biennial CBE screening (age 40-69) with treatment of stage I to IV; (13) Biennial mammography screening (age 40-69) with Stage I-IV treatment; (17) Biennial mammography screening (age 50-69) with EPC and stage I-IV treatment
**Peregrino, 2012** [44]	Brazil (Upper MIC)	Model based	CEA	Women aged 50 years and higher in Brazil	(1) no screening; (2) conventional film mammography; (3) digital mammography and (4) magnetic resonance imaging.
**González-Mariño, 2012** [45]	Colombia (Upper MIC)	Model based	CEA	Women aged 50-69 years old in Bogota, Colombia	Mammography screening VS no screening. Screening group has four coverage’s alternatives (20% 40% 60% and 80%)
**Valencia-Mendoza, 2009** [46]	Mexico (Upper MIC)	Model based	CEA	Women aged 24-99 years old in Mexico	13 alternative mammography screening programs with age-difference to start the screening program (at 40, 48 or 50 years), with two levels of coverage (25%, and 50%) and two frequency periods of screening (every year, or every two years): (1) 40, 25, 1 (2) 48, 25, 1 (3) 50, 25, 1 (4) 40, 25, 2 (5) 48, 25, 2 (6) 50, 25, 2 (7) 40, 50, 1 (8) 48, 50, 1 (9) 50, 50, 1 (10) 40, 50, 2 (11) 48, 50, 2 (12) 50, 50, 2 (13) no screening
**Okonkwo, 2008** [47]	India (Lower MIC)	Model based	CEA	Women aged 40-70 years old in India	(1) One lifetime CBE age 50; (2) One lifetime CBE age 40; (3) 5 years interval CBE age 50-70; (4) One lifetime MMG age 50; (5) 5 years interval CBE age 40-60; (6) One lifetime MMG age 40; (7) Biennial CBE age 50-70; (8) Biennial CBE 40-60 (base); (9) Annual CBE age 40-60; (10) Biennial MMG age 50-70; (11) Biennial MMG age 40-60
**Groot, 2006** [48]	Africa (Upper MIC, Lower MIC, and LIC)	Model based	CEA	Women aged 50-70 years old in Africa	Six strategies: (1) Stage I treatment; (2) Stage II treatment; (3) Stage III treatment; (4) Stage IV treatment; (5) Treatment all stages; (6) Extensive program (treatment of all stages plus a breast awareness program and early case finding through biannual mammographic screening)

CEA – cost-effectiveness analysis, CUA – cost-utility analysis, MIC – middle-income countries, LIC – low-income countries, CBE – clinical beast examination, MAR – mass media awareness raising, EPC – extended palliative care, NMPOA – *Nucleo Mama Porto Alegre*, BSMP – *Bahçeşehir* Mammography Screening Project, TNBCRP – Turkish National Breast Cancer Registry Program, CBE – clinical breast examination, MMG – mammography, US – ultrasonography, SFM – screen-film mammography, FFDM – full-field digital mammography

Reviewed studies were based on either mathematical models (n = 18) or empirical data (n = 3). Most studies (67%) combined costs and effects in cost-effectiveness analyses (CEA), while the other studies were cost-utility analyses (CUA). **Table 2** presents the intervention evaluated by each study. A total of thirteen studies were evaluating mammography screening alone, while eight studies evaluated mammography screening in combination with some other breast cancer control. From those eight studies, five studies combined mammography screening with treatment strategies for breast cancer patients stages I-IV; two studies combined mammography screening with risk factor assessment and ultrasound or clinical breast examination (CBE).

**Table 3** presents further study characteristics. Most studies used a lifetime horizon or something approximating lifetime (n = 18); other studies used 10 years (n = 1), one year (n = 1) or were unclear about the time horizon used (n = 1). Furthermore, only four studies were conducted using a societal perspective. In contrast, the other studies used a health care system perspective (n = 12), or a third-party payer perspective (n = 1). The perspectives in the remaining four studies were not clearly stated. In addition, most studies applied discounting for both costs and effects (n = 14), whereas two studies applied discounting to the costs only, three studies did not apply discounting at all, two studies provided unclear information, none applied differential discounting.

**Table 3:** Characteristics of reviewed studies and sensitivity analyses conducted

Authors	Time horizon	Perspective	Discount rate	Probabilistic sensitivity analysis	One-way sensivity analysis	Scenario analysis	Model validation
**Wang, 2021** [28]	Lifetime	Society	5% (cost and effect)	No	Yes	Yes	Yes
**Salikhanov, 2019** [29]	Lifetime	Health care	4.8% (cost)	No	Yes	No	No
**Sun, 2018** [30]	Lifetime	Society	3% (cost and effect)	Yes	Yes	Yes	No^*^
**Nguyen, 2018** [31]	Lifetime	Health care	3% (cost and effect)	Yes	Yes	Yes	No
**Wu, 2017** [32]	Lifetime	Unclear	No discounting*	No	Yes	No	No
**Ozmen, 2017** [33]	Lifetime	Society	Unclear	No	Yes	Yes	No
**Zehtab, 2016** [34]	Unclear	Third party payer (insurance)	3% (cost)	No	Yes	No	No
**Haghighat, 2016** [35]	Lifetime	Health Care	5% (cost) and 3% (effect)	Yes	Yes	No	No
**Ulloa-Pérez, 2016** [36]	10 years	Unclear	Unclear	No	No	Yes	No
**Barfar,2014** [37]	1 year	Health care	No discounting	No	No	Yes	No
**Nie ¨ns, 2014** [38]	Lifetime	Health care	3% (cost and effect)	No	Yes	No	No
**Souza, 2013** [39]	Lifetime	Health care	5% (cost and effect)	Yes	Yes	Yes	Yes
**Zelle, 2013** [40]	Lifetime	Health care	3% (cost and effect)	No	Yes	No	No
**Ribeiro, 2013** [41]	Lifetime	Health Care	5% (cost and effect)	No	Yes	No	No
**Ginsberg, 2012** [42]	Lifetime	Unclear	3% (cost and effect)	Yes	No	No	No
**Zelle, 2012** [43]	Lifetime	Health care	3% (cost and effect)	No	Yes	No	No
**Peregrino, 2012** [44]	Lifetime	Health Care	Unclear	No	Yes	No	No
**González-Mariño, 2012** [45]	Lifetime	Health Care	No discounting	Yes	Yes	Yes	Yes
**Valencia-Mendoza, 2009** [46]	Lifetime	Health Care	3% (cost and effect)	Yes	Yes	Yes	Yes
**Okonkwo, 2008** [47]	Lifetime	Unclear	3% (cost and effect)	No	Yes	No	Yes
**Groot, 2006** [48]	Lifetime	Society	3% (cost and effect)	No	Yes	Yes	No

QALY – quality adjusted life year, DALY – disability adjusted life year, LYG –life years gained

*This study did a calibration, but we did not consider it as a full validation.

**Table 3** shows that most of the included studies applied one-way sensitivity analysis, with several of them also applying probabilistic sensitivity analysis or scenario analysis. However, only three studies explicitly validated the model they used for their analysis. **Table 4** presents detailed results of ICER and ACER for each study.

**Table 4:** Results of cost and effectiveness of reviewed studies

Authors	Effectiveness outcome measure	Incremental effectiveness		Currency and year	Incremental costs	ACER	ICER	Conclusion by authors (quality of studies according to CHEC)
**Wang, 2021** [28]	LYG	7963 per 100 000 women		US$ (2019)	79 100 000	17 309 (screening)	25 261 (biennial 45-70 years old); 24 138 (triennial 45-70 years old); 14 437 (triennial 50-70 years old)	MMG was shown to be cost-effective ***(good)***
**Salikhanov, 2019** [29]	QALY	790		US$ (2016)	2 500 000	1113 (screening); 1001 (non-screening)	3157	MMG was shown to be cost-effective ***(moderate)***
**Sun, 2018** [30]	QALY	Screening vs no screening	Annual = 0.0286; Every 3 years = 0.0127; Every 5 years = 0.0076	US$ (2014)	Annual = 235.76; Every 3 years = 84.99; Every 5 = years 52.41	Annual = 8243; Every 3 years = 6692; Every 5 years = 6896	Annual = 8253; Every 3 years = 6671; Every 5 years = 6917	MMG in combination with other strategies was shown to be cost-effective, but MMG alone is not cost-effective ***(good)***
		MMG vs MMG+US	Annual = -0.0014; Every 3 years = -0.0011; Every 5 years = -0.0007		Annual = -29.02; Every 3 years = -11.73; Every 5 years = -6.72	Annual = 13.32; Every 3 years = 7.52; Every = 5 years 6.32	Annual = 21 246; Every 3 years = 11 000; Every 5 years = 9366	
**Nguyen, 2018** [31]	LYG	120 (age 45-49); 289 (age 50-54); 239 (age 55-59); 163 (age 60-64) - per 100 000 women		US$ (unclear year)	1 054 339 (age 45-49); 1 053 353 (age 50-54); 1 049 332 (age 55-59); 1 031 089 (age 60-64)	*Screening* = 11.63 (age 45-49); 11.53 (age 50-54); 11.50 (age 55-59); 11.21 (age 60-64) *Non-screening*: 1.15 (age 45-49); 1.04 (age 50-54); 1.00 (age 55-59); 0.80 (age 60-64)	8782 (45-49 years old); 3647 (50-54 years old); 4405.44 (55-59 years old); 6335 (60-64 years old)	MMG was shown to be cost-effective in the age groups of age 50±54 and age 55±59, but not cost-effective in age 45±49 and age 60±64 ***(moderate)***
**Wu, 2017** [32]	QALY	CBE + US-MMG = 1206; US-MMG = 1241; CBE-US-MMG = 1313		RMB (CNY) ¥ (unclear year)	CBE + US-MMG = 174.13 million; US-MMG = 227.66 million; CBE-US-MMG = 1175.37 million	Unclear	CBE + US-MMG = 144 386; US-MMG = 183 449; CBE-US-MMG = 895 179	MMG in combination with other strategies was shown to be cost-effective ***(moderate)***
**Ozmen, 2017** [33]	LYG	4.17		US$ (2014)	677 171	71 205 (BSMP); 50 802 (TNBCRP)	2423	MMG in combination with other strategies was shown to be cost-effective ***(good)***
**Zehtab, 2016** [34]	DALY	0.05307		US$ (2013)	-332.34	77 082.5 (screening); 589 027 (non-screening)*	-6264	MMG was shown to be cost-effective ***(moderate)***
**Haghighat, 2016** [35]	QALY	0.007 (first round); 0.003 (second round); 0.001 (third round)		Int $ (unclear year)	249 (first round); 355 (second round); 551 (third round)	266.3 (first round); 397.9 (second round); 621.2 (third round)	37 350 (first round); 141 641 (second round); 389 148 (third round)	MMG was shown to be cost-effective for the first round of triennially mammography screening, but not for the second and third rounds ***(moderate)***
**Ulloa-Pérez, 2016** [36]	DALY	Unclear		$ Mexican Pesos (2015)	Unclear	10 741 (current scenario); 448 (feasible scenario); 12 696 (objective scenario)	Unclear	MMG was shown to be cost-effective ***(low)***
**Barfar,2014** [37]	Number of detected cancer case	24 per 100 000 women		US$ (2008)	Unclear	Unclear	15 742	MMG was not shown to be cost-effective ***(moderate)***
**Niëns, 2014** [38]	DALY	*Costa Rica* =1102 (1); 634 (12); 259 (13); 1279 (15). *Mexico* = 32 908 (14); 11 284 (10); 3424 (11); 6382 (13)		US$ (2009)	*Costa Rica* = 5 222 329 (1); 8 509 853 (12); 7 854 300 (13); 7 629 450 (15). *Mexico* = 165 249 320 (14); 145 182 285 (10); 47 912 496 (11); 109 230 958 (13)	*Costa Rica* = 4739 (1); 7085 (12); 8924 (13); 5397 (15). *Mexico* = 7025 (10); 7526 (11); 8659 (13); 5021 (14)	*Costa Rica* = 4739 (1); 13 426 (12); 30 352 (13); 5964 (15); other strategies were dominated. *Mexico* = 12 718 (10); 13 994 (11); 17 115 (13); 5021 (14); other strategies were dominated	MMG in combination with other strategies was shown to be cost-effective ***(good)***
**Souza, 2013** [39]	QALY	34 (Strategy C); 14 (Strategy B); 3 (Strategy F)		Brazillian Real (2010)	50 (Strategy C); 193 (Strategy B); 75 (Strategy F)	6.8 (Strategy C); 6.3 (Strategy B); 6.1 (Strategy F)	1509 (Strategy C); 13 131 (Strategy B); 30 520 (Strategy F); Other strategies were dominated.	MMG was shown to be cost-effective ***(good)***
**Zelle, 2013** [40]	DALY	3.78 (59); 0.11 (65); 0.07 (67); 0.30 (94)		US$ (2012)	15 611 (59); 599 (65); 1937 (67); 9925 (94)	4125 (67); 4167 (65); 4582 (59); 6595 (94)	4125 (67); 5659 (65); 27 477 (59); 87 423 (94); other strategies were dominated	MMG in combination with other strategies was shown to be cost-effective ***(good)***
**Ribeiro, 2013** [41]	QALY	13.97 (A); 14.05 (B)		Brazillian Real (2010 and 2011)	1107 (B)	18 667 (B)	13 426 (B)	MMG in combination with other strategies was shown to be cost-effective ***(good)***
**Ginsberg, 2012** [42]	DALY	*Sub-Saharan Africa* = 313 (6); 188 (12); 94 (18) – per 1 000 000 population *South East Asia* = 201 (6); 120 (12); 60 (18) – per 1 000 000 population		Int $ (2005)	*Sub-Saharan Africa* = 0.68 (6); 0.41 (12); 0.25 (18). *South East Asia* = 0.87 (6); 0.53 (12); 0.28 (18)	*Sub-Saharan Africa* = 2248 (6); 2253 (12); 2323 (18). *South East Asia* = 4338 (6); 4362 (12); 4399 (18)	*Sub-Saharan Africa* = 2248 (6); 2261 (12); 2696 (18). *South East Asia* = 4338 (6); 4401 (12); 4596 (18).	MMG in combination with other strategies was shown to be cost-effective***(good)***
**Zelle, 2012** [43]	DALY	12 560 (11); 2020 (13); 1.00 (17)		US$ (2009)	16 311 046 (11); 26 081 188 (13); 548 459 (17)	1299 (11); 2907 (13); 2945 (17)	1299 (11); 12 908 (13); 553 616 (17); other strategies were dominated	MMG was not shown to be cost-effective ***(good)***
**Peregrino, 2012** [44]	LYG	0.178 (2); 0.016 (4)		Brazillian Real (unclear year)	2 412 769.97 (2); 46 007 401.6 (4)	5588.00 (1); 74 627.00 (2); 174 127.00 (3); 1 390 998.00 (4)	13 573.07 (2); 2 904 328.88 (4); other strategies were dominated	MMG was shown to be cost-effective***(low)***
**González-Mariño, 2012** [45]	LYG	6979 (cycle 20)		Colombian Peso (2004)	2 000 000 (cycle 20)	Unclear	7791.34 US dollars for 2.8 GDP, and 8069.6 US dollars for 2.9 GDP	MMG was shown to be cost-effective ***(low)***
**Valencia-Mendoza, 2009** [46]	LYG	241 826, 782 534, 1 152 898 (strategy 5, 10, 7)		Mexican Peso (2007)	18 200 000, 91 100 000, 197 300 000 (subsequently for the first, second, third option)	Unclear	75 000 pesos for 0.7 GDP (strategy 5), 116 000 pesos for 1.6 GDP (strategy 7), 171 000 pesos for 1.6 GDP (strategy 7)	MMG was shown to be cost-effective ***(moderate)***
**Okonkwo, 2008** [47]	LYG	1837 (Strategy 5); 2434 (Strategy 8); 2436 (Strategy 9); 713 (Strategy 11) – per 1 000 000 women		Int $ (2001)	2 300 000 (Strategy 5); 3 800 000 (Strategy 8); 7 300 000 (Strategy 9); 13 700 000 (Strategy 11)	1137 (Strategy 5); 1348 (Strategy 8); 1919 (Strategy 9); 3469 (Strategy 11)	1251 (Strategy 5); 1549 (Strategy 8); 3108 (Strategy 9); 19 257 (Strategy 11); Other strategies were dominated.	MMG was not shown to be cost-effective***(good)***
**Groot, 2006** [48]	DALY	Unclear		US$ (2000)	Unclear	78 (1); 324 (2); 389 (3); 4986 (4); 159 (5); 75 (6)	75 (6)	MMG in combination with other strategies was shown to be cost-effective***(good)***

ICER – incremental cost-effectiveness ratio, ACER – average cost-effectiveness ratio, CBE – clinical breast examination, MMG – mammography, US – ultrasonography, BSMP – *Bahçeşehir* Mammography Screening Project, TNBCRP – Turkish National Breast Cancer Registry Program, GDP – gross domestic product

*The results presented by the author in the narrative and table were switched. However, reading through the paper, we finally use the number being given in the narrative as it was making more sense.

**Table 4** shows the results of cost and effectiveness of reviewed studies. Nine studies used base years of cost data from 2010 onwards; eight studies before 2010; while it was unclear what the remaining four studies used. Nearly all studies expressed final health outcomes: QALY (n = 6), DALY (n = 7), and LY (n = 7). Only one study used an intermediate health outcome concerning the number of detected cancer cases.

### Study findings

**Table 4** also presents the study findings of each reviewed study. 18 from 21 studies concluded that mammography screening, whether or not in combination with other strategies, is cost-effective.

Among 13 studies comparing mammography screening alone to no-screening or another screening strategy, 11 studies stated that mammography screening is cost-effective. However, in two of these 11 studies, there are pre-requisites to achieving cost-effectiveness. More specifically, Nguyen et al. [31] stated that mammography screening would be cost-effective if the program was conducted in women aged 50-54 years or 55-59 years. On the other hand, the program was not cost-effective for women aged 45-49 years or 60-64 years. Haghighat et al. [35] stated that the first round of mammography screening is cost-effective while the second and third rounds are not, due to lower detection rates.

Among eight studies which evaluated mammography screening in combination with other strategies for breast cancer control, seven studies concluded that mammography screening is cost-effective. However, two of these seven studies concluded that mammography alone is not a cost-effective strategy. Furthermore, one study considered mammography screening in combination with other strategies for breast cancer control as not cost-effective.

To optimally assess cost-effectiveness, screening frequency and population age need to be considered. Studies included in this review evaluated multiple age groups, but mostly (80%) from the age between 40 and 70 years. They compared a range of screening frequencies (from annually to triennially). The studies by Ginsberg et al. (50-70 years) [42], Gonzalles-Marino (50-69 years) [45], Niëns et al. (40-70 years and 50-70 years) [38], and Ozmen et al. (40-69 years) [33] used biennial mammography screening for their program and deemed it cost-effective. Furthermore, Souza et al. [39] revealed that biennial screen-filmed mammography is more economically viable than annual screening (both strategies were cost-effective). Wang et al. [28] found that biennial mammography (45-70 years) is more cost-effective than triennial mammography (45-70 years). Ulloa-Perez et al. [36] reported that mammography screening approaches cost-saving only if the screening interval is biennial or triennial (40-70 years).

In terms of the type of mammography device, two studies made a comparison between conventional (screen-filmed) mammography and digital mammography [39,44]. Both studies concluded that conventional mammography is more cost-effective compared to digital mammography. This was mainly due to the higher cost of digital mammography. However, Souza et al. [39] also revealed that digital mammography could be cost-effective if implemented in the <50 years old (the age group where digital mammography has the highest benefit) in combination with conventional mammography in the 50-69 years age group.

Coverage rate was also evaluated by studies included in this review. Wang et al. [28] evaluated coverage of 100%, 80%, or 60%; Ginsberg et al. [42] assessed coverage of 50%, 80%, or 90%; Gonzales Marino et al. [45] used coverage of larger than 50%; Valencia-Mendoza et al. [46] evaluated coverage of 25% and 50%. The results were varied as presented in **Table 4****.**

#### Risk of bias assessment

**Figure 2:**
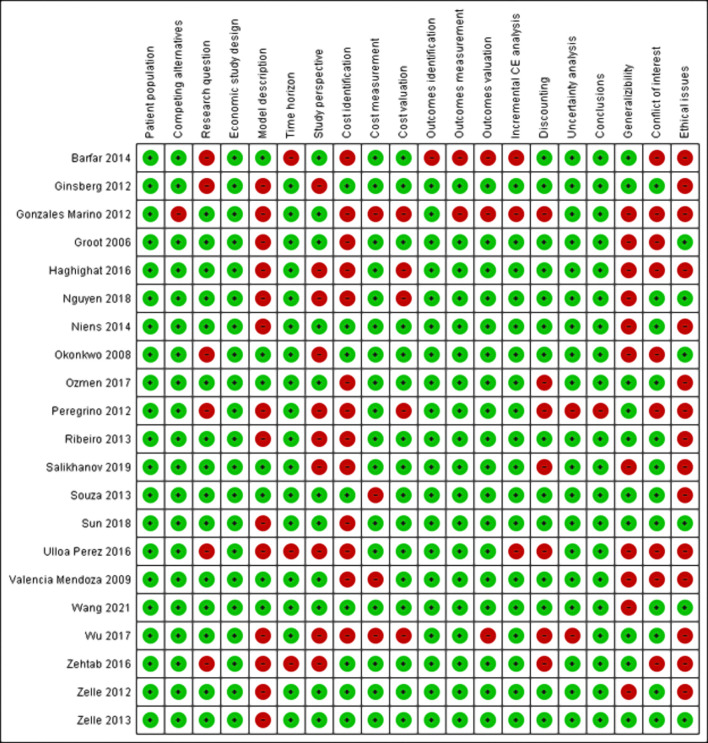
Results of risk of bias assessment using CHEC-extended checklist.

**Figure 2** and **Table 5** summarize the results of risk of bias assessment of included studies, as indicated by the percentage score. Each item was scored 1 (green light) if the study met the requirement or 0 (red light) if it did not meet or only partly met the requirements. Moreover, we noted “not applicable” (N/A) if the item assessed was not relevant for that particular study and it was scored as 1. The quality of all studies ranged from 40% to 95%. 11 studies were categorized as good quality [28,30,33,38,39-43,47,48], seven as moderate quality [29,31,32,34,35,46], and three as low quality [36,44,45].

**Table 5:** Quality assessment of reviewed studies using CHEC-extended checklist, ordered by year publication

Authors	1	2	3	4	5	6	7	8	9	10	11	12	13	14	15	16	17	18	19	20	Score	Grade
**Wang, 2021** [28]	1	1	1	1	1	1	1	1	1	1	1	1	1	1	1	1	1	0	1	1	95%	Good
**Salikhanov, 2019** [29]	1	1	1	1	NA (1)	1	0	0	1	1	1	1	1	1	0	1	1	0	1	0	75%	Moderate
**Sun, 2018** [30]	1	1	1	1	0	1	1	0	1	1	1	1	1	1	1	1	1	1	1	1	90%	Good
**Nguyen, 2018** [31]	1	1	1	1	0	1	0	0	1	0	1	1	1	1	1	1	1	0	1	1	75%	Moderate
**Wu, 2017** [32]	1	1	1	1	0	1	0	0	0	0	1	1	0	1	0	0	1	1	1	0	55%	Moderate
**Ozmen, 2017** [33]	1	1	1	1	NA (1)	1	1	0	1	1	1	1	1	1	0	1	1	1	1	0	85%	Good
**Zehtab, 2016** [34]	1	1	0	1	0	0	0	1	1	1	1	1	1	1	0	1	1	1	0	0	65%	Moderate
**Haghighat, 2016** [35]	1	1	1	1	0	1	0	0	1	0	1	1	1	1	1	1	1	0	0	0	65%	Moderate
**Ulloa-Pérez, 2016** [36]	1	1	0	1	0	0	0	0	1	1	1	1	1	0	0	1	1	0	0	0	50%	Low
**Barfar, 2014** [37]	1	1	0	1	NA (1)	0	1	0	1	1	0	0	0	0	1	1	1	1	0	0	55%	Moderate
**Nie ¨ns, 2014** [38]	1	1	1	1	0	1	1	1	1	1	1	1	1	1	1	1	1	0	1	0	85%	Good
**Souza, 2013** [39]	1	1	1	1	1	1	1	1	0	1	1	1	1	1	1	1	1	1	1	0	90%	Good
**Zelle, 2013** [40]	1	1	1	1	0	1	1	1	1	1	1	1	1	1	1	1	1	1	1	1	95%	Good
**Ribeiro, 2013** [41]	1	1	1	1	0	1	0	0	1	1	1	1	1	1	1	1	1	1	1	0	80%	Good
**Ginsberg, 2012** [42]	1	1	0	1	0	1	0	1	1	1	1	1	1	1	1	1	1	1	1	0	80%	Good
**Zelle, 2012** [43]	1	1	1	1	0	1	1	1	1	1	1	1	1	1	1	1	1	0	1	0	85%	Good
**Peregrino, 2012** [44]	1	1	0	1	0	1	0	0	1	0	1	1	1	1	0	0	0	1	0	0	50%	Low
**González-Mariño, 2012** [45]	1	0	1	1	0	1	1	0	0	0	1	0	0	0	0	1	1	0	0	0	40%	Low
**Valencia-Mendoza, 2009** [46]	1	1	1	1	1	1	1	0	0	1	1	1	1	1	1	1	1	0	0	0	75%	Moderate
**Okonkwo, 2008** [47]	1	1	0	1	1	1	0	1	1	1	1	1	1	1	1	1	1	0	0	1	80%	Good
**Groot, 2006** [48]	1	1	1	1	0	1	1	0	1	1	1	1	1	1	1	1	1	0	0	1	80%	Good

Studies generally (70%) scored 0 on item number 5 as they mainly lacked stating assumptions made in their model or in model validation. Furthermore, most studies (75%) scored 0 on item number 20 since they did not sufficiently discuss ethical and distributional issues. Half of the studies scored 0 on items number 7, number 8, and number 18. Those studies did not justify not using a societal perspective, not accounting for all important and relevant cost (eg, follow-up or palliative cost), or not discussing the generalizability of their results to other settings and patient/client groups.

**Table 4** shows the conclusions made by each study and the study quality. Among 18 studies which concluded that mammography screening is cost-effective, with or without combination with other strategies, study quality varied from low to good. In detail, nine studies have good quality, seven studies have moderate quality, and two studies have low quality. On the other hand, from three studies that concluded that mammography screening is not cost-effective, two have good quality and one has moderate quality.

## DISCUSSION

This review identified 21 studies on the economic evaluation of mammography as a breast cancer screening method in LMICs. 19 studies were from MICs (Upper or Lower) and two studies from both MICs and LICs. By conducting this systematic review in 2021, we found 17 additional studies examining the cost-effectiveness of mammography screening than the 2013 systematic review by Zelle et al. [49] about economic evaluation of breast cancer control in LMICs. Our systematic review found that mammography screening appeared to be a cost-effective strategy, particularly in Upper MICs.

### Study findings and implications

18 out of 21 studies included in our review concluded that mammography screening was a cost-effective strategy, some of which did not assess mammography as a single screening strategy compared to no screening. Instead, 12 studies compared mammography screening only to no strategy or other strategy; two studies compared the combination of mammography, risk-based assessment, and ultrasound/CBE to no strategy or other strategy; four studies compared mammography screening plus treatment of stage I-IV to other strategy.

The majority of those 18 studies concluding that mammography screening was a cost-effective strategy was conducted in Upper MICs (71%). This conclusion appears to be in line with the WHO guideline, which states that an organized population-based mammography screening is recommended for implementation in well-resourced settings or limited resource settings with relatively strong health systems [1].

The majority (76%) of the included studies in our review specified the screening interval. For most of those studies the screening interval was two years, which was in general found to be cost-effective. When a comparison was made between biennial screening and annual or triennial screening, a biennial interval was found to be more cost-effective than the other screening interval strategies. This is in line with the conclusion of the review of systematic reviews by Mandrik et al. [18] as well as the WHO recommendation of a screening interval of two years among women aged 50-69 years in well-resourced settings or limited resource settings with relatively strong health systems [1]. This recommendation was based on modelling studies and further analysis of trials showed that screening every two years seems to provide the best trade-off between benefits (mortality reduction) and harms (overdiagnosis or overtreatment).

It is also essential to discuss the issue of the appropriate age group for screening. In several HICs (eg, United States, Sweden, Japan) mammography screening begins at the age of 40 years [50,51]. However, there is clear uncertainty about the magnitude of overdiagnosis among both younger and older women. Mammography screening at age 40 years reduced mortality more than at age 50, but it consumed more resources and resulted in more false-positives [1,49]. This also occurs in screening among women over 69 years of age that will generate some mortality reduction, but will also substantially increase overdiagnosis [1].

Generally, the studies included in our review examined mammography screening among women aged 40-70 years. Five studies explored mammography in the range of 50-70 years old and concluded that mammography screening is cost-effective. In contrast, 15 studies assessed mammography screening at a younger starting age (ie, 25, 35, 40, 45 years old). These studies came up with inconclusive results on its cost-effectiveness. Although the target age group is a major issue in organizing a breast cancer screening program, each country has its specific incidence and unique screening design which cannot be easily adopted from different settings [52].

Two of the studies included in our review also examined women by a risk-based approach. The screening strategy evaluated by Ribeiro et al. [41] combined mammography screening, CBE, and risk factor assessment. Sun et al. [30] used ‘Your Disease Risk’ which calculates individual cancer risk, after which high-risk women underwent mammography and ultrasound screening. The economic analysis by Ribeiro et al [41] and Sun et al. [30] revealed that screening with a risk-based approach is cost-effective. Some researchers argued that risk-based screening might not be optimal, considering that 80% of women with newly diagnosed breast cancers have no known major risk factors [50,53]. However, compared to one-size-fits-all screening strategy that may induce unnecessary inclusion of a large population with lower risk, screening individuals tailored to higher risk of developing breast cancer could be a more economically viable alternative [52].

The most recent study in China (Upper MIC) revealed that biennial mammography screening compared to no-screening cost US$25 261 per life years gained, which was considered cost-effective [28]. Furthermore, the budget impact analysis showed that screening would result in a net cost of US$38.1 million for a city with one million citizens for ten years. On the other hand, a study from sub-Saharan African (Lower MIC) and Southeast Asia countries (LIC) showed that biennial mammography screening in combination with treatment of all cancer stages cost Int$2000-6000 per DALY averted and can also be considered cost-effective [48]. To our knowledge, budget impact analysis studies on mammography screening in these regions are still unavailable. Thus, it is difficult to estimate the affordability of mammography screening program in Lower MIC and LIC.

### Quality of evidence

We found 18 studies that concluded mammography screening is cost-effective, with or without combination with other strategies, with studies’ quality varying from low to good. Three studies that concluded that mammography screening is not cost-effective were of good and moderate quality. This variation complicates the grading of the strength of evidence and advocating a recommendation. However, if we ignore the item in CHEC which would not influence the results of ICER (ie, discussion on ethics, distributional, and generalizability, conflict of interest), the majority of studies (80%) that concluded mammography screening is cost-effective would be rated as good, while the quality rating of studies which conclude that mammography screening is not cost-effective remain the same.

Nearly all studies with good quality were model-based. It is generally advised to the use of modelling studies in economic analysis. Modelling approaches enable the researcher to be flexible in the inclusion of important methodological characteristics such as adequate time horizon and more appropriate to evaluate a broad array of interventions across different groups [49]. However, most studies (70%) got a zero score concerning model validation in their research. Model validation is important in showing if the model sufficiently represents the system under assessment. The lack of model validation may have influenced the ICER results and therefore affect the extent to which results can serve as a solid basis for decision making [54].

### Strengths and limitations

One strength of our review was that it captured a substantial amount of full economic evaluation studies (CEA or CUA) in LMICs, as we used a comprehensive search strategy and included studies without any language or year’s limitation. This review included 17 additional studies conducted in LMICs compared to another published systematic review on breast cancer control [49]. However, most studies were conducted in Upper MICs, and only few studies were from Lower MICs or LICs.

We did not assess publication bias in this review. This may lead to an overestimation on the cost-effectiveness of mammography. To minimize the publication bias, we also searched at Embase database, which covered grey literature such as conference proceedings [55].

Furthermore, there are some concerns raised by Welch et al. [56] worth mentioning here although they may not apply in the same way to LMICs. First, mammography screening may cause overdiagnosis (mostly of small tumours) and therefore overestimation of the benefit of screening. Second, reduction in fatality rates over time may be a result of improved treatment options. Both these issues could lead to overly optimistic assessments of cost-effectiveness. Although a model-based cost-effectiveness analysis would ideally adjust for these things, there may still be confounding in the background, and so results should be interpreted with caution.

We used the CHEC-extended checklist, which can appraise model-based or trial-based or observational-based cost-effectiveness studies. However, this checklist is quite generic to accommodate studies of various methodologies. Some of the CHEC items only apply to model-based analyses and it is complicated to rate the risk of bias of an observational-based study according to these same standards. Therefore risk of bias scores are difficult to compare across studies and should be interpreted with caution.

Drawing an overall conclusion for a systematic review of economic evaluation has always been a difficult task. A quantitative meta-analysis of ICERs, costs, or health benefits is hardly feasible due to heterogeneity across study designs, methods, country policies, health care settings, and many further practical challenges [57]. Therefore, the synthesis of results commonly takes a narrative approach, as we applied in this review. Without pooling the results, narrative synthesis could identify outcome patterns relating to the direction of an effect [58]. In addition, determining the transferability of the findings of this review to other countries remains a challenge.

## CONCLUSIONS

To conclude, this systematic review found that mammography screening was a cost-effective strategy in LMICs, particularly in Upper MICs. However, there is still limited evidence in the Lower MICs and LICs and the quality of studies varied widely. Thus, more studies conducted in Lower MICs and LICs are needed to better understand the cost-efectiveness of mammography screening in these regions.
